# Depression, Anxiety, and Stress Symptoms in Women with Rheumatic Disease of Reproductive Age: Lessons from the COVID-19 Pandemic

**DOI:** 10.3390/jcm14145038

**Published:** 2025-07-16

**Authors:** Nora Rosenberg, Antonia Mazzucato-Puchner, Peter Mandl, Valentin Ritschl, Tanja Stamm, Klara Rosta

**Affiliations:** 1 Department of Obstetrics and Gynaecology, Clinical Division of Gynaecological Endocrinology and Reproductive Medicine, Medical University of Vienna, 1090 Vienna, Austria; nora.rosenberg@meduniwien.ac.at; 2 Department of Psychiatry and Psychotherapy, Clinical Division of Social Psychiatry, Medical University of Vienna, 1090 Vienna, Austria; 3 Department of Internal Medicine III, Division of Rheumatology, Medical University of Vienna, 1090 Vienna, Austria; 4 Centre for Medical Data Science, Institute for Outcomes Research, Medical University of Vienna, 1090 Vienna, Austria; 5 Ludwig Boltzmann Institute for Arthritis and Rehabilitation, 1090 Vienna, Austria

**Keywords:** mental health, systemic autoimmune rheumatic disease, anxiety, depression, stress, coronavirus

## Abstract

**Background:** Women with systemic autoimmune rheumatic disease (SARD) are at higher risk of developing infection-related complications, anxiety, and depression. Using the example of the COVID-19 pandemic, we aimed to explore the impact of this external stressor on symptoms of depression, anxiety, and stress in a sample of women with SARD in a cross-sectional study design. **Methods:** Females aged 18–50 with SARD were enrolled from 04/2021 to 04/2022 at the Medical University of Vienna or through an online self-help group, while snowball sampling was used to recruit an age-matched healthy control group. Participants completed questionnaires including: (1) demographic information, medical history, and access to healthcare; (2) the Depression, Anxiety, and Stress Scale (DASS-21); and (3) the Coronavirus Anxiety Scale (CAS). Parameters were compared between groups using Chi-squared, Fisher’s exact, and Mann–Whitney U tests. Linear regression analysis was used to investigate which individual factors predicted the DASS-21 in women with SARD. **Results:** The study sample consisted of 226 women (n = 99 with SARD and n = 127 healthy controls). Women with SARD reported lower DASS-21 stress (*p* = 0.008) and CAS scores (*p* = 0.057) than the control group. There were no significant differences in DASS-21 anxiety or depression scores. Among women with SARD, a linear regression model identified the most important predictors of DASS-21 as access to rheumatological care (*p* = 0.002) and recent disease activity (*p* = 0.028). **Conclusions:** Despite the pandemic, women with SARD reported mental health outcomes equal to or better than those of the healthy control group. Continued access to rheumatological care may serve as an important protective factor for their mental health during large-scale crises like pandemics.

## 1. Introduction

Women have twice the risk of developing depression [1] and anxiety disorders [2] across their lifespan and up to four times the risk of developing autoimmune diseases, including systemic autoimmune rheumatic diseases (SARD), compared with men [3]. SARD affects about 3% of women of reproductive age [4]. Alongside genetic and environmental factors, sex hormone fluctuations seem to exert an important role in the development of anxiety [5], depression [6], and SARD [7]; phases of hormonal transition like adolescence, pregnancy, the post-partum period, and peri-menopause are often associated with their onset and can influence their course [8,9,10]. SARD is linked to additional risk for developing depression and anxiety [11], which has been attributed to pain, disability, and direct effects of inflammation on the brain [12].

Numerous studies reported that the beginning of the COVID-19 pandemic was associated with a significant reduction in well-being and a rise in anxiety and depression symptoms in the general population [13,14]. Women and people with pre-existing somatic diseases were particularly affected [15] and more likely to experience health-related anxiety during this period [16]. The rise of anxiety and depression symptoms in women was attributed to increased psychosocial stress. This included balancing home-office with responsibilities like home-schooling, difficulties obtaining childcare, and disruptions to employment and family income [17]. Women were also more likely to lose their jobs during the pandemic [18] and experienced a significant rise in domestic abuse and violence [19]. Mental health symptoms in people with chronic diseases were attributed to their presumed vulnerability to severe COVID-19 infection and the anxiety and stress caused by healthcare disruptions [20]. Other studies detected more COVID-19 specific fear and distress in people with chronic diseases, without differences in anxiety and depression symptoms compared with the general population [21,22] and a large multinational study observed resilience in one third of a sample with chronic diseases [23]. Resilience refers to the capacity of an individual or group to adapt and preserve mental health during challenging or stressful circumstances [24,25,26]. It can be either measured through resilience questionnaires (e.g., measuring specific traits or coping skills) [23], which tend to have poor predictive utility for mental health outcomes [25,27], or through the analysis of mental health trajectories after exposure to adversity [23]. Individuals are considered resilient when they exhibit few psychological symptoms (such as anxiety, stress, or depression) in response to a stressful event over time [23].

Only a few studies have examined the mental health of people with SARD during the pandemic or previous historic crises, and those studies have reported contradictory results. Two cross-sectional studies [28,29] suggested higher levels of anxiety and depression in patients with SARD compared with healthy controls at the beginning of the COVID-19 pandemic, while concurrent cross-sectional studies reported a similar incidence of mental health problems between patients with SARD and healthy controls [30,31]. A longitudinal study [32] observed increased distress among patients with SARD compared with pre-pandemic data, but no increase in mental health problems. Data on patients with SARD during other large-scale crises is scarce. To our knowledge, the only study on this topic [33] found that patients with rheumatoid arthritis were more likely to experience depressive symptoms and increased disease activity after the San Francisco earthquake or Hurricane Hugo. So far, no studies on large scale crises have focused on women of reproductive age with SARD who face multiple risk factors for anxiety and depression.

In the present study, our primary aim was to investigate the effect of the COVID-19 pandemic on symptoms of depression, anxiety, stress, and coronavirus anxiety in women with SARD of reproductive age compared with a healthy control group. We hypothesised that women with SARD would score higher than healthy controls for these psychological symptoms. Our secondary aim was to identify predictors of depression, anxiety, and stress symptoms in the SARD group. We hypothesised that healthcare access, comorbidities, organ involvement, activity, or treatment of the underlying rheumatic disease and coronavirus anxiety would predict greater levels of depression, anxiety, and stress in women with SARD.

## 2. Materials and Methods

### 2.1. Study Design and Data Collection

This cross-sectional questionnaire study collected data between April 2021 and April 2022. The recruitment of women with SARD occurred primarily at the Medical University of Vienna, in the “Rhe-Pro” (rheumatic disease and reproduction) outpatient clinic. An online questionnaire link (www.soscisurvey.de, accessed on 8 April 2021) was additionally sent to the Austrian support group for patients with SARD “Selbsthilfegruppe der Österreichischen Rheumaliga”. For the recruitment of healthy control subjects, the questionnaire link was distributed through snow-ball sampling. On the first page of the survey, all participants were provided with a disclaimer detailing this study’s purpose, their rights, the assurance of anonymity, and a consent statement with a tick box. Consent was given by ticking the box. The questionnaires are included in the Supplementary Information. This study was conducted following the Declaration of Helsinki and good clinical practice guidelines and was approved by the ethical committee of the Medical University of Vienna (reference number: 2399/2020, date of approval 7 April 2021). This study was conducted in accordance with the STROBE checklist for cross-sectional studies.

### 2.2. Participants

Inclusion criteria for the SARD group were women aged 18–50 with previously diagnosed SARD. The latter inclusion criterion was ensured by recruiting patients with SARD who were either waiting for their appointment at the Rhe-Pro outpatient clinic or were members of a self-help group for patients with SARD. SARD diagnoses included inflammatory joint diseases, systemic connective tissue diseases, and vasculitis. An overview of specific diagnostic subgroups represented in the sample can be found in Table 2. The control group had the same age criteria but without a SARD diagnosis. Exclusion criteria for both groups were severe self-reported co-morbidities (e.g., chronic obstructive pulmonary disease, cardiac insufficiency, active malignancy) and insufficient proficiency in German.

### 2.3. Measures

Participants were asked to complete a self-composed questionnaire featuring questions on demography, health, and access to medical services during the pandemic (see Supplementary Information, S1). Participants were asked about their sexual orientation, and no statistical difference was found between groups. In addition, they filled the Depression, Anxiety and Stress Scale (DASS-21), a validated screening device with 21 items for these symptoms [34] (see Supplementary Information, S2). Nilges & Essau [35] validated a German version of the DASS-21, available without a license, which has also been validated in chronic pain patients. Through a 4-point Likert scale, participants select the degree to which different statements applied to them in the last week. The questionnaire measures depression, anxiety, and stress with 7 subscale items for each domain. Subscale scores are summed for each domain and for the composite DASS-21 score. Finally, we collected information using the Coronavirus Anxiety Scale (CAS), a validated screening tool for dysfunctional anxiety about the coronavirus with five items [36] (see Supplementary Information, S3). It can be used without a license and has been validated in German [37]. The single scale measure assesses such anxiety during the last two weeks. Participants indicate how much each statement applies to them on a 5-point Likert scale. The CAS score is calculated by summing the item responses.

### 2.4. Statistical Analysis

Descriptive results were presented separately for both groups. Differences between variables were assessed using the Chi-square, followed by Fisher’s exact and Mann–Whitney U test as appropriate. The Bonferroni–Holm method was utilised to correct for multiple testing. A linear regression model was performed to test which individual factors predicted the composite DASS-21 score. Predictors were selected based on the literature and clinical expertise. Multicollinearity was assessed through variance inflation factors (VIFs). The distribution of the model’s residuals was examined through a histogram, a P-P Plot, and a scatterplot of predicted vs. residual values. Supplementary sensitivity analyses using linear regressions models were used to adjust for potential confounders. IBM SPSS Statistics (Version 27) was used for data analysis, and RStudio (Version 2024.09.1+394) for visualisation. 

## 3. Results

### 3.1. Descriptive Statistics

#### 3.1.1. Demographic Data

**Figure 1:**
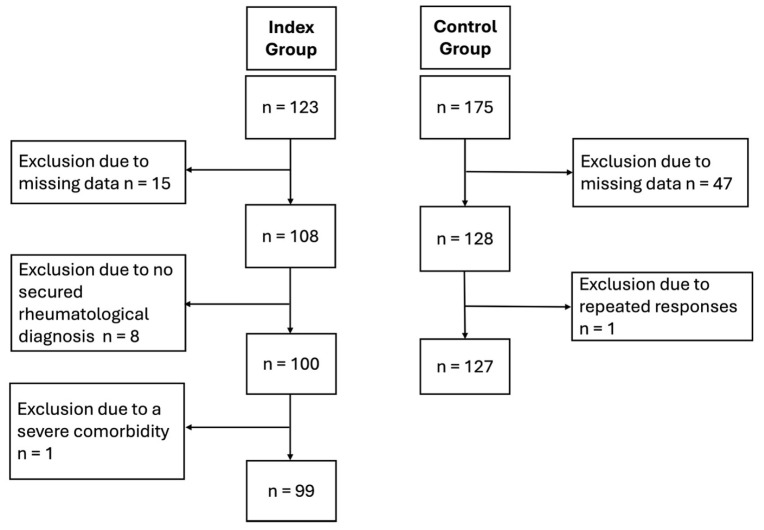
Inclusion and exclusion flowchart.

n = 123 participants were enrolled in the SARD group, and n = 175 in the control group. In the SARD group, n = 24 participants had to be excluded due to incomplete data, no secured rheumatic diagnosis, or a severe co-morbidity. In the control group, n = 48 participants were excluded due to missing data or duplicate open-end responses, indicating that they completed the questionnaire twice. n = 99 participants remained in the SARD and n = 127 in the control group. Figure 1 describes the inclusion and exclusion process.

The participants’ age range was normally distributed between 18–20 and 46–50 years, with 31–35 being the most frequent interval; 29 participants in the SARD group (29.3%) and 40 in the control group (31.5%) were in this age range (Table 1). No significant differences in age intervals were found between the two groups (*p* = 0.448). Regarding nationality, 77 participants in the SARD group were Austrian (77.8%) versus 89 participants (70.1%) in the control group (*p* = 0.193). There were no significant differences between groups in employment status (*p* = 0.608), financial change experienced due to the COVID-19 pandemic (*p* = 0.452), relationship status (*p* = 0.681), or COVID-19 infection status in the past year (*p* = 0.191).

Women in the SARD group were significantly more likely to report an internal long-term condition other than SARD (*p* < 0.001). Specifically, 24 participants (24.2%) in the SARD group reported having one or more of these conditions, in contrast to 9 participants (7.1%) in the control group.

#### 3.1.2. Rheumatic Disease Characteristics

Within the SARD group, 80 participants (80.8%) indicated having a single SARD, while 19 individuals (19.1%) indicated two or more. Connective tissue disease (CTD) was the most common category, with 57 participants (57.6%) having one or more of these diagnoses. Inflammatory joint disease (IJD) was the second most represented category, with 46 participants (46.5%) having one or more of these diagnoses. Vasculitis was the least represented group, with four participants (4.0%). For more details on SARD characteristics, see Table 2. 

Fifty-one participants (51.5%) indicated that organs beyond the musculoskeletal system were affected by their rheumatic disease. Fifty participants (50.5%) reported disease activity within the last six months. Eighty-five participants (85.9%) stated they were currently taking antirheumatic medication: fifty (50.5%) took conventional disease-modifying antirheumatic drugs (cDMARDs), thirty-five (35.4%) took biologic disease-modifying antirheumatic drugs (bDMARDs), and ten (10.1%) took glucocorticoids. Eighteen participants (18.1%) reported difficulties accessing rheumatology care during the pandemic, while seventy-seven participants (77.8%) reported no problem, and four participants (4.0%) with SARD did not seek consultation.

### 3.2. Mental Health Variables

**Figure 2:**
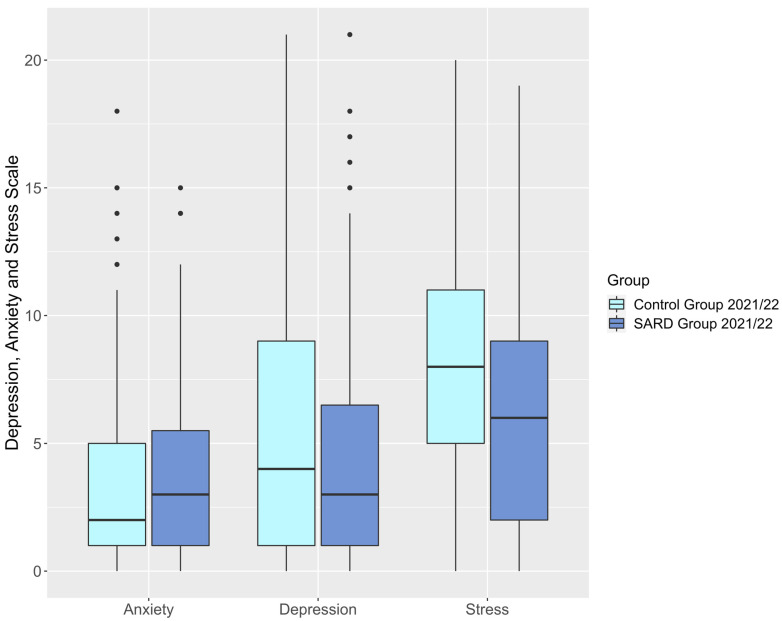
Boxplot, DASS-21 scores of control group vs. SARD group in 2021/22.

**Figure 3:**
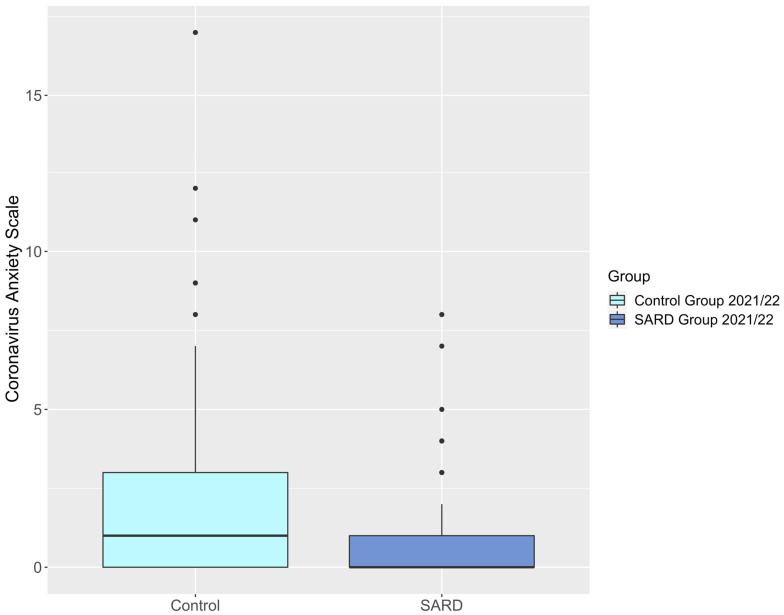
Boxplot, CAS scores of control group vs. SARD group in 2021/22.

The SARD group had significantly lower stress scores than the control group (SARD vs. C 6.37 ± 5.05 vs. 8.39 ± 5.00, *p* = 0.008) with 95% confidence intervals (CI) at [5.37, 7.38] for SARD and [7.51, 9.26] for controls. There were no significant differences between the two groups in anxiety and depression symptoms. The SARD group exhibited lower scores on the CAS than the control group (SARD vs. C 1.09 ± 1.76 vs. 1.91 ± 2.81, *p* = 0.057) with 95% CI at [0.74, 1.44] and [1.42, 2.41], respectively. However, this difference did not survive corrections for multiple testing. Table 3 and Figure 2 and Figure 3 display results that compare the DASS-21 and the CAS between the SARD and the control group. Supplementary sensitivity analyses (see Supplementary Information, S4, models 1 and 2) were performed using linear regression models to adjust for differences in comorbidities between the two groups. After controlling for internal chronic diseases other than SARD, having a SARD diagnosis remained a significant independent predictor of both DASS-21 (ß= −0.14 *p* = 0.045 at 95% CI [−6.77,−0.08]) and CAS scores (ß= −0.17 *p* = 0.014 at 95% CI [−1.47,−0.17]). Further sensitivity analyses were performed to adjust for COVID-19 infection status in the past year (see Supplementary Information, S4, models 5 and 6), which did not exert a significant influence on either DASS-21 or CAS scores.

#### Linear Regression Model

The model predicted 22% of variance in the total DASS-21 score. Two variables were significant predictors—difficulties obtaining a rheumatological appointment during the pandemic had the strongest impact (β = 0.31; *p* = 0.002) with 95% CI [4.01, 16.98], followed by disease activity within the last six months (β = 0.22; *p* = 0.031) with 95% CI [0.59, 10.28]. See Table 4 for more details.

Supplementary subgroup analyses for participants with inflammatory joint diseases (IJD) and systemic connective tissue diseases (SCTD) (see Supplementary Information, S4, models 3 and 4, respectively) revealed that access to rheumatological appointments predicted the most variance in the total DASS-21 score in both groups, with ß = 37, *p* = 0.01 at 95% CI [3.18, 22.34] in the IJD group and ß = 30, *p* = 0.033 at 95% CI [3.18, 22.34] in the SCTD group. In the IJD group, having another comorbidity also asserted a significant influence on the total DASS-21 score (ß = 29, *p* = 0.042 at 95% CI [0.27, 14.37]).

## 4. Discussion

Contrary to our hypothesis, the SARD group showed significantly lower DASS-21 stress scores than the control group, with no significant differences in depression or anxiety scores. As hypothesised, access to rheumatology care and recent disease activity were significant predictors of the overall DASS-21 score among women with SARD during the pandemic.

Our finding that the SARD group appeared less stressed and exhibited lower coronavirus anxiety contrasts with studies from early 2020, which reported higher DASS-21 scores, more COVID-19 peritraumatic distress [28,29], and more worry and stress [32] in patients with SARD compared with healthy controls. This discrepancy may be due to age differences, as these studies included individuals of advanced age, which has been linked to lower well-being [32]. Other studies with younger participants found no significant differences between patients with SARD and controls [30,31]. Our study is consistent with Seyahi et al.’s findings [31] on anxiety and depression and is the first to report better stress and coronavirus anxiety outcomes in patients with SARD compared with healthy controls.

Potential explanations for our finding include prior experience of patients with SARD coping with chronic illness, less disruption of everyday life due to already limited mobility and social exposure, access to specialised medical care, and gender specific factors. Firstly, living with the psychological and physical impairments of SARD may be a “training camp” for coping with stressful events like a pandemic [38] and people with autoimmune conditions may previously have had to develop coping strategies when facing isolation due to disease factors or treatment effects [39]. Secondly, the conditions of the pandemic and lockdown may have been perceived as “levelling” between people with chronic diseases and the general population since the restrictions affected everybody and worries about health became more prevalent and socially accepted than before [40]. Thirdly, people with chronic inflammatory diseases in relatively affluent developed countries like Austria tend to have more contact with healthcare professionals than people in the general population. This may have given the SARD group an “advantage” over healthy women in terms of better access to the latest medical information, which in turn might have reduced stress and coronavirus anxiety. Fourthly, although female gender has been identified as a predictor of psychological distress during the pandemic in the general population, none of the studies comparing individuals with SARD to a healthy control group have identified female gender as a risk factor for adverse mental health outcomes during the pandemic [28,29,31,32]. Ciaffi et al. [38]’s study on patients with inflammatory arthritis during the pandemic found that women with inflammatory arthritis had significantly higher resilience scores than healthy controls, but this effect could not be replicated for males. Capuano et al.’s study [40] of patients with MS observed an improvement in quality of life and sexual satisfaction scores during the pandemic (compared with before), remaining significant for female but not for male participants after correction procedures. These findings, alongside our results, suggest that female gender may be a protective factor in populations with autoimmune conditions during a pandemic.

Our finding that difficulty obtaining a rheumatological appointment was the most important predictor of the composite DASS-21 score highlights the importance of stable healthcare access for mental health outcomes during a pandemic. This variable explained more variance in psychological outcomes than recent disease activity, coronavirus anxiety, comorbidities, antirheumatic medication, or organ involvement. Fortunately, only 18% of the SARD group faced difficulties obtaining an appointment, but this minority suggests that healthcare disruption can increase depression, anxiety, and stress symptoms in people with chronic diseases. This finding is consistent with a multi-centre study on patients with SARD in fifteen Arab countries [41], where 73% reported the pandemic had impacted their mental health, which was primarily associated with reduced access to rheumatological appointments, alongside struggles with medication persistency and accessing hydroxychloroquine. In a Mexican study [42], 52.5% of patients with SARD reported healthcare interruption, which was associated with a lower quality of life. In addition to the tangible consequences of healthcare disruptions, such as reduced access to medication and medical consultations, the mere knowledge of stable healthcare access may also play an important protective role in mitigating psychological distress during a pandemic. This aligns with a large body of the literature on stress and support, particularly the “stress buffering hypothesis” [23], which suggests that the perceived availability of support can alleviate the impact of stressors [43].

Consistent with prior research on mental health in patients with SARD [44,45], recent disease activity in our sample predicted the overall DASS-21 score. This association may reflect the psychological toll of persistent pain and fatigue, disease-specific symptoms such as swollen joints, heightened negative affect, and elevated proinflammatory cytokines [46]. Comorbid conditions emerged as an important predictor of elevated DASS-21 scores within the IJD subgroup. This aligns with findings by Marrie et al. [47], who observed that physical comorbidities increased the risk of depression and anxiety in patients with RA.

The present study is the first to investigate psychological outcomes in women of reproductive age with SARD in response to a large-scale crisis. However, limitations include potential bias through self-report measurements, lack of psychiatric comorbidity assessment, and the cross-sectional design. This study’s exclusive reliance on self-report measures may impact the reliability of medical information, specifically the diagnostic classification of rheumatic diseases and comorbidities. However, this approach ensured privacy when collecting data on sensitive topics such as mental health. Although subjects were asked whether they suffer from (other) internal diseases, we did not specifically assess for psychiatric comorbidities or the intake of psychotropic medications. This is a limitation since both factors could be confounding variables, potentially moderating or mediating the assessed mental health outcomes. The cross-sectional assessment of the main outcomes without pre-pandemic mental health baseline data makes it unclear how far inter-group differences can be attributed to the pandemic. Within these measures, more valid inferences about the pandemic can be drawn from measures referring directly to the pandemic (CAS and access to health services) rather than the DASS-21 scale. The timing of the survey was both a strength and limitation. Unlike other studies, the present study did not capture effects of the first lockdown in the spring of 2020. However, it offered a more long-term insight into women’s lives during later stages of the pandemic in 2021 and 2022.

Future research should aim to replicate and expand these findings using prospective longitudinal study designs to assess changes in mental health over time and in response to ongoing stressors. Incorporating validated resilience measures and qualitative assessments could provide more insight into underlying psychological mechanisms. Evaluating the impact of specific healthcare aspects, such as telemedicine availability, could clarify the role of healthcare access for mental health outcomes. Future studies could feature larger samples with a broader age range and participants of all genders to allow for the exploration of gender- and age-specific mechanisms and to increase the generalisability of findings. A better understanding of resilience in patients with SARD, including its development and characteristics, could be used to strengthen their resilience in a clinical context.

## 5. Conclusions

Despite the pandemic, women of reproductive age with SARD reported mental health outcomes that were equal to or better than those of a healthy control group. The exploration of potential risk and resilience factors indicates that stable access to healthcare might play a protective role for the mental health of this population during a large-scale crisis. Given the challenges the pandemic posed to in-person care, these results raise the question of whether alternative models, such as telemedicine, could offer similar mental health benefits by maintaining continuity of care when traditional access is limited.

## Figures and Tables

**Table 1:** Demographic variables.

Variable	SARD Group (n = 99) n (%)	Control Group (n = 127) n (%)	*p*-Value
**Age**			0.448 ^a^
18–20	1 (1.0)	3 (2.4)	
21–25	13 (13.1)	9 (7.1)	
26–30	21 (21.2)	21 (16.5)	
31–35	29 (29.3)	40 (31.5)	
36–40	23 (23.2)	34 (26.8)	
41–45	5 (5.1)	13 (10.2)	
46–50	7 (7.1)	7 (5.5)	
Missing data	0	0	
**Nationalities**			0.193 ^a^
Austrian	77 (77.8)	89 (70.1)	
Others	22 (22.2)	38 (29.9)	
Missing data	0	0	
**Employment Status**			0.608 ^b^
In full-time education	4 (4.0)	11 (8.7)	
Working	71 (71.7)	86 (67.7)	
Pregnancy/parental leave	15 (15.2)	22 (17.3)	
Unemployed	4 (4.0)	4 (3.1)	
Others (paid leave/ health leave/housewife)	5 (5.1)	4 (3.1)	
Missing data	0	0	
**Change in economic situation due to COVID**			452 ^b^
No change	74 (74.7)	86 (67.7)	
I do not know	7 (7.1)	5 (3.9)	
Positive change in economic situation	5 (5.1)	12 (9.4)	
Reduction monthly income 1–30%	10 (10.1)	15 (11.8)	
Reduction monthly income 31–50%	2 (2.0)	7 (5.5)	
Reduction monthly income 51–70%	1 (1.0)	2 (1.6)	
Missing data	0	0	
**Relationship Status**			0.681 ^a^
Single	9 (9.1)	18 (14.2)	
In a relationship < 1 year	4 (4.0)	6 (4.7)	
In a relationship > 1 year	46 (46.5)	54 (42.5)	
Married	40 (40.4)	49 (38.6)	
Missing data	0	0	
**Chronic disease (other than SARD)**			<0.001 ***^a^
Yes	24 (24.2)	9 (7.1)	
No	75 (75.8)	118 (92.9)	
Missing data	0	0	
**COVID-19 infection in the past year**			0.191
Yes	14 (14.1)	10 (7.8)	
No	85 (85.8)	117 (92.1)	
Missing data	0	0	

Note. *** *p* < 0.001, ^a^ Chi-square test, ^b^ Fisher’s exact test.

**Table 2:** Rheumatic disease parameters.

Variable	n (%)
**Number of Rheumatic Diagnoses**	
1	80 (80.8)
2	17 (17.1)
3	2 (2.0)
**Inflammatory Joint Disease (IJD) ^a^**	46 (46.5)
Rheumatoid Arthritis (RA)	31 (31.3)
Ankylosing Spondylitis (AS)/ Non-Radiographic Axial Spondylitis (nr-axSpA)	10 (10.1)
Psoriatic Arthritis (PsA)	7 (7.1)
Juvenile Idiopathic Arthritis (JIA)	1 (1.0)
**Systemic Connective Tissue Disease (SCTD) ^a^**	57 (57.6)
Systemic Lupus Erythematosus (SLE)	41 (41.4)
Sjögren’s Syndrome (SS)	14 (14.1)
Mixed or Undifferentiated Connective Tissue Disease (MCTD)/(UCTD)	5 (5.0)
Antiphospholipid Syndrome (APLAS)	3 (3.0)
Systemic Sclerosis (SSc)	2 (2.0)
**Vasculitis ^a^**	4 (4.0)
Behçet’s Disease	4 (1.0)
Relapsing Polychondritis	2 (2.0)
Microscopic Polyangiitis	1 (1.0)
**Organ Involvement**	
Yes	51 (51.5)
No	48 (48.5)
**Disease Activity in the Last 6 Months?**	
Yes	50 (50.5)
No	49 (49.5)
**Currently on Antirheumatic Medication?**	
Yes	85 (85.9)
No	14 (14.1)
**Type of Medication**	
Conventional Disease-Modifying Antirheumatic Drug (cDMARD)	50 (50.5)
Biologic Disease-Modifying Antirheumatic Drug (bDMARD)	35 (35.4)
Glucocorticoid	10 (10.1)
**Difficulties Accessing Rheumatology Care During COVID-19 Pandemic?**	
Yes	18 (18.1)
No	77 (77.8)
Did not seek Consultation	4 (4.0)

Note. ^a^ denotes that there was more than one possible answering option. No data were missing for the reported rheumatic disease parameters.

**Table 3:** Mental health variables in SARD and control group during the pandemic.

Variable	SARD Group 2021/22 (n = 99) Mean ± SD; Median (Range) 95% CI (Lower, Upper)	Control Group 2021/22 (n = 127) Mean ± SD; Median (Range) 95% CI (Lower, Upper)	*p*-Value	Corrected *p*-Value
DASS 21-Depression Score	4.63 ± 5.03; 3.00 (0–21) 3.63, 5.64	5.61 ± 5.32; 4.00 (0–21) 4.68, 6.55	0.128	0.256
DASS 21-Anxiety Score	3.87 ± 3.79; 3.00 (0–15) 3.11, 4.63	3.59 ± 3.95; 2.00 (0–18) 2.9, 4.28	0.437	0.437
DASS 21-Stress Score	6.37 ± 5.05; 6.00 (0–19) 5.37, 7.38	8.39 ± 5.00; 8.00 (0–20) 7.51, 9.26	0.002 **	0.008 **
Coronavirus Anxiety Scale	1.09 ± 1.76; 0.00 (0–8) 0.74, 1.44	1.91 ± 2.81; 1.00 (0–17) 1.42, 2.41	0.019 *	0.057

Note. Comparisons were made using the Mann–Whitney U Test. * *p* < 0.05; ** *p* < 0.01. DASS-21= Depression, Anxiety, and Stress Scale with 21 items.

**Table 4:** Linear regression model predicting the total DASS-21 score in the SARD group.

Predictors	Unstandardised Coefficient (B)	Standard Error	Standardised Coefficient (β)	95% CI (Lower, Upper)
Difficulties obtaining a rheumatological appointment during the pandemic (no vs. yes)	10.50	3.27	0.31 **	4.01, 16.98
Currently taking antirheumatic medication (yes vs. no)	4.22	3.58	0.12	−2.89, 11.32
Organ involvement (no vs. yes)	1.38	2.43	0.06	−3.44, 6.21
Active disease within the last 6 months (no vs. yes)	5.44	2.44	0.22 *	0.59, 10.28
Other internal chronic disease (no vs. yes)	4.28	2.91	0.15	−1.51, 10.07
Coronavirus anxiety score	1.35	0.70	0.19	−0.05, 2.74

Note. n = 97. R^2^ = 0.22 (adjusted R^2^= 0.16) and the F for the change in R^2^ is 4.13 (*p* < 0.001). * *p* < 0.05, ** *p*< 0.005.

## Data Availability

The data supporting this study’s results are available from the corresponding author upon reasonable request.

## Supplementary Materials

The following supporting information can be downloaded at https://www.mdpi.com/article/10.3390/jcm14145038/s1. S1: Self-composed questionnaire on demography, medical history and healthcare access during the pandemic (in German). S2: Depression, Anxiety and Stress Scale with 21 Items (in German). S3: Coronavirus Anxiety Scale (in German). S4: Sensitivity Analyses.

## Abbreviations

The following abbreviations are used in this manuscript:

APLAS	Antiphospholipid Syndrome
AS	Ankylosing Spondylitis
axSpA	Axial Spondylarthritis
bDMARDs	Biologic Disease-Modifying Antirheumatic Drugs
CAS	Coronavirus Anxiety Scale
cDMARDs	Conventional Disease-Modifying Antirheumatic Drugs
DASS-21	Depression, Anxiety and Stress Scale with 21 Items
JIA	Juvenile Idiopathic Arthritis
MCTD	Mixed Connective Tissue Disease
PsA	Psoriasis Arthritis
RA	Rheumatoid Arthritis
Rhe-Pro	Rheumatic Disease and Reproduction
SLE	Systemic Lupus Erythematosus
SpA	Spondylarthritis
SS	Sjögren’s Syndrome
SSc	Systemic Sclerosis
STROBE	Strengthening The Reporting of Observational Studies in Epidemiology
UCTD	Undifferentiated Connective Tissue Disease
VIF	Variance Inflation Factor
