# Supplementary material for: Depression, Anxiety, and Stress Symptoms in Women with Rheumatic Disease of Reproductive Age: Lessons from the COVID-19 Pandemic
Source: J Clin Med. 2025 Jul 16;14(14):5038. doi: 10.3390/jcm14145038 (PMC12295548; doi:10.3390/jcm14145038)
Supplement: Supplementary file 1 [file jcm-14-05038-s001.zip › jcm-3717592-supplementary.pdf]

# S1: Self-composed questionnaire on demography, medical history and healthcare access during the pandemic (in German)

Sehr geehrte Teilnehmerinnen!

Im Rahmen meiner Diplomarbeit untersuche ich, welche Auswirkungen die COVID-19 Pandemie auf Sexualität, Familienplanung und psychisches Befinden bei Frauen mit rheumatischen Erkrankungen zwischen 18 und 50 Jahren hat. Ihre Teilnahme leistet einen wertvollen Beitrag zu dieser Forschung.

Das Ausfüllen des Fragebogens ist freiwillig und dauert etwa 30 Minuten. Die Datenerhebung erfolgt vollständig anonym. Es ist für niemanden möglich, von den Fragebögen auf die Identität der jeweiligen Teilnehmerin zu schließen. Sie können jederzeit aufzuhören, den Fragebogen auszufüllen.

Bei Fragen können Sie sich gerne an das Studienteam wenden:

- Dr. Klara Rosta (Geburtshilfe, Studienärztin), klara.rosta@meduniwien.ac.at
- Dr. Antonia Puchner (Rheumatologie, Studienärztin) antonia.puchner@meduniwien.ac.at
- Nora Rosenberg (Diplomandin), n11715669@students.meduniwien.ac.at

☐ Ich erkläre mich damit einverstanden, an diesem Fragebogen teilzunehmen und erteile die Erlaubnis, dass die von mir im Zuge der Umfrage erhobenen Daten zu Forschungszwecken verwendet werden dürfen.

## Demographie:

**1) Wie alt sind Sie?**

□ 18-20

□ 21-25

□ 26-30

□ 31-35

□ 36-40

□ 41-45

□ 46-50

**2) Welche Nationalität(en) haben Sie?**______________________________________________

**3) In welchem Bundesland leben Sie?**

| | | | | |
|---|---|---|---|---|
| □ Wien | □ Niederösterreich | □ Burgenland | □ Steiermark | □ Salzburg |
| □ Oberösterreich | □ Kärnten | □ Tirol | □ Vorarlberg | □ Ich wohne nicht in Österreich. |

**4) Derzeitige Tätigkeit:** □ Schülerin Schulrichtung: _____________________

□ Studentin Studienrichtung: ____________________

□ Berufstätigkeit Berufszweig: _____________________

□ Karenz □ Arbeitslos

□ Sonstige:______________

**5) Wie führen Sie diese Tätigkeit aktuell überwiegend durch?**

□ Homeoffice

□ Distance Learning (Schule/Studium/Ausbildung)

□ Physische Anwesenheit am Arbeitsplatz

□ Abwechselnd Homeoffice und Arbeitsplatz

□ Ich habe durch die Pandemie meine vorherige Arbeit verloren

□ Ich war auch schon vor der Pandemie nicht erwerbstätig

**6) Hat COVID Ihre wirtschaftliche Lage verändert?**

□ Nein □ Ich weiß nicht

□ Ja, COVID hat meine wirtschaftliche Lage begünstigt

Ja, Reduktion des monatlichen Einkommens um:

□ 1-30%

□ 31-50%

□ 51-70%

□ 71-100%

**7) Höchster Abschluss:** □ Hauptschule □ Berufsschule □ Matura

□ Bachelor □ Master/Mag. □ Doktor

□ Sonstige: _____________________

8) **Beziehungsstatus:** □ ledig □ in einer Beziehung (< 1 Jahr)

□ verheiratet □ in einer Beziehung (> 1 Jahr)

9) **Sexuelle Orientierung** □ heterosexuell □ bisexuell □ homosexuell

□ asexuell □ andere

10) **Aktuelle Wohnsituation:** □ alleine □ mit Partner/Partnerin □ mit Eltern

□ mit Kind/Kindern

□ mit Partner/Partnerin und Kind/Kindern

□ mit Eltern, Partner/Partnerin und Kind/Kindern

□ Wohnheim □ WG □ Andere ____________________

11) **Wie viele Personen leben insgesamt aktuell in Ihrem Haushalt**?

□ 1 □ 2 □ 3 □ 4 □ 5 □ 6 □ 7 □ 8 oder mehr

## Krankengeschichte/Anamnese:

1) **Hatten Sie im vergangenen Jahr eine COVID-19 Infektion?** □ Ja □ Nein

2) **Ist bei Ihnen eine internistische Erkrankung/Grunderkrankung bekannt?** □ Ja □ Nein

**Wenn ja, welche?**

| | | |
|---|---|---|
| ☐ Lungenerkrankung | ☐ Rücken-/Gelenkserkrankung | ☐ Stoffwechselerkrankung |
| ☐ Blase/Niere | ☐ Blut-/Gerinnungserkrankung | ☐ Neurologische Erkrankung |
| ☐ Magen-/Darmerkrankung | ☐ Hauterkrankung | ☐ Krebserkrankung |
| ☐ Herz-/Kreislauferkrankung | ☐ Sonstige: | |

**Genauer Name Ihrer Erkrankung:**________________________________

3) **Leiden Sie an einer rheumatischen Erkrankung?** □ Ja □ Nein
**Wenn ja, an welcher?**

| | | | |
|---|---|---|---|
| □ Rheumatoide Arthritis | □ Spondylarthritis | □ Sjögren-Syndrom | □ Systemische Vaskulitis |

| | | | |
|---|---|---|---|
| □ Antiphospholipid-Syndrom | □ Systemischer Lupus erythematodes | □ Systemsklerose | □ Sonstige: |

4) **War im Rahmen Ihrer Erkrankung jemals ein anderes Organsystem betroffen?** □ Ja □ Nein

**Wenn ja, welches?** □ Haut □ Nervensystem □ Niere □ Leber □ Magen-Darm-Trakt □ Sonstige:___________

5) **Seit wann ist Ihnen Ihre Erkrankung bekannt (Erstdiagnose)?** ______________________

6) **Hatten Sie in den letzten 6 Monaten eine ärztlich bestätigte rheumatische Krankheitsaktivität?**
□ Ja □ Nein

7) **Welche Medikamente nehmen Sie aufgrund Ihrer rheumatischen Erkrankung?** □ Keine

| | | | |
|---|---|---|---|
| □ Arava | □ Benlysta | □ CellCept/Myfortic | □ Cimzia |
| □ Enbrel | □ Endoxan | □ Humira | □ Ilaris |
| □ Immunosporin/Cicloral | □ Imurek/Immunoprin | □ Kineret | □ MabThera |
| □ Methotrexat/Ebetrexat | □ Orencia | □ Plaquenil/Quensyl | □ Prograf |
| □ Remicade | □ RoActemra | □ Salazopyrin | □ Simponi |
| □ Stelara | □ Tauredon | □ Sonstige:__________ | |

8) **Wann war Ihre letzte Kontrolle bei Ihrem Rheumatologen/Internisten bzw. Ihrer Rheumatologin/ Internistin?**

□ vor 1-3 Monaten □ vor 4- 6 Monaten
□ vor mehr als 6 Monaten □ vor über einem Jahr

9) **Hatten sie während der COVID Pandemie Schwierigkeiten einen Termin bei Ihrem Rheumatologen/Internisten bzw. Ihrer Rheumatologin/ Internistin zu bekommen**?

□ ja □ nein □ Ich habe es nicht versucht

10) **Hatten sie während der COVID Pandemie Schwierigkeiten Zugang zu Ihrer Behandlung zu bekommen**?

□ ja □ nein □ Ich habe es nicht versucht

11) **Rauchen Sie?** □ Ja □ Nein
**Wenn ja, wie viele Zigaretten rauchen Sie pro Tag?** ______

12) **Wie oft trinken Sie Alkohol?** □Täglich □ Wöchentlich □ Monatlich □ Nie

## S2: Depression, Anxiety and Stress Scale with 21 Items (in German)

Bearbeitungshinweis: Bitte lesen Sie jede Aussage und kreuzen Sie die Zahl 0, 1, 2 oder 3 an, die angeben soll**, wie sehr die Aussage während der letzten Woche auf Sie zutraf**. Es gibt keine richtigen oder falschen Antworten. Versuchen Sie, sich spontan für eine Antwort zu entscheiden.
0 Traf gar nicht auf mich zu
1 Traf bis zu einem gewissen Grad auf mich zu oder manchmal
2 Traf in beträchtlichem Maße auf mich zu oder ziemlich oft
3 Traf sehr stark auf mich zu oder die meiste Zeit

| | | | | |
|---|---|---|---|---|
| 1. Ich fand es schwer, mich zu beruhigen. | 0 | 1 | 2 | 3 |
| 2. Ich spürte, dass mein Mund trocken war. | 0 | 1 | 2 | 3 |
| 3. Ich konnte überhaupt keine positiven Gefühle mehr erleben. | 0 | 1 | 2 | 3 |
| 4. Ich hatte Atemprobleme (z. B. übermäßig schnelles Atmen, Atemlosigkeit ohne körperliche Anstrengung) | 0 | 1 | 2 | 3 |
| 5. Es fiel mir schwer, mich dazu aufzuraffen, Dinge zu erledigen. | 0 | 1 | 2 | 3 |
| 6. Ich neigte dazu, auf Situationen überzureagieren. | 0 | 1 | 2 | 3 |
| 7. Ich zitterte (z. B. an den Händen). | 0 | 1 | 2 | 3 |
| 8. Ich fand alles anstrengend. | 0 | 1 | 2 | 3 |
| 9. Ich machte mir Sorgen über Situationen, in denen ich in Panik geraten und mich lächerlich machen könnte. | 0 | 1 | 2 | 3 |
| 10. Ich hatte das Gefühl, dass ich mich auf nichts mehr freuen konnte. | 0 | 1 | 2 | 3 |
| 11. Ich bemerkte, dass ich mich schnell aufregte. | 0 | 1 | 2 | 3 |
| 12. Ich fand es schwierig, mich zu entspannen. | 0 | 1 | 2 | 3 |
| 13. Ich fühlte mich niedergeschlagen und traurig. | 0 | 1 | 2 | 3 |
| 14. Ich reagierte ungehalten auf alles, was mich davon abhielt, meine momentane Tätigkeit fortzuführen. | 0 | 1 | 2 | 3 |
| 15. Ich fühlte mich einer Panik nahe. | 0 | 1 | 2 | 3 |
| 16. Ich war nicht in der Lage, mich für irgendetwas zu begeistern. | 0 | 1 | 2 | 3 |
| 17. Ich fühlte mich als Person nicht viel wert. | 0 | 1 | 2 | 3 |
| 18. Ich fand mich ziemlich empfindlich. | 0 | 1 | 2 | 3 |
| 19. Ich habe meinen Herzschlag gespürt, ohne dass ich mich körperlich angestrengt hatte (z. B. Gefühl von Herzrasen oder Herzstolpern). | 0 | 1 | 2 | 3 |
| 20. Ich fühlte mich grundlos ängstlich. | 0 | 1 | 2 | 3 |
| 21. Ich empfand das Leben als sinnlos | 0 | 1 | 2 | 3 |

## S3: Coronavirus Anxiety Scale (in German)

| **Wie oft haben Sie in den letzten 2 Wochen Folgendes erlebt?** | Überhaupt nicht | Selten, weniger als 1-2 Tage | Mehrere Tage | Mehr als 7 Tage | Nahezu täglich in den letzten 2 Wochen |
|---|---|---|---|---|---|
| **1. Ich fühlte mich schwindelig, benommen oder matt, wenn ich Nachrichten über das Corona-Virus las oder hörte.** | ☐ | ☐ | ☐ | ☐ | ☐ |
| **2. Ich hatte Schwierigkeiten ein- oder durchzuschlafen, weil ich über das Corona-Virus nachgedacht habe.** | ☐ | ☐ | ☐ | ☐ | ☐ |
| **3. Ich habe mich gelähmt oder wie erstarrt gefühlt, wenn ich an das Corona-Virus dachte oder Informationen darüber ausgesetzt war.** | ☐ | ☐ | ☐ | ☐ | ☐ |
| **4. Ich verlor das Interesse am Essen, wenn ich an das Corona-Virus dachte oder Informationen darüber ausgesetzt war.** | ☐ | ☐ | ☐ | ☐ | ☐ |
| **5. Mir wurde übel oder ich hatte Magenprobleme, wenn ich an das Corona-Virus dachte oder Informationen darüber ausgesetzt war.** | ☐ | ☐ | ☐ | ☐ | ☐ |

# S4: Sensitivity Analyses

1. **Comorbidities**

**Model 1: Linear Regression Model predicting the total DASS-21 Score with SARD group membership and comorbidities as predictors**

| Predictors | Unstandardised coefficient (B) | Standard Error | Standardised coefficient (β) | 95% CI (lower, upper) |
|---|---|---|---|---|
| SARD (no vs. yes) | -3.42 | 1.70 | -.14* | -6.77,-.08 |
| Other internal chronic disease (no vs. yes) | 6.01 | 2.37 | .17* | 1.34,10.67 |

**Note.** n = 222. $R^2$ = .038 (Adjusted $R^2$= .029) and the F for the change in $R^2$ is 4.27 (p=.015). *p<0.05.

**Model 2: Linear Regression Model predicting the total CAS Score with SARD group membership and comorbidities as predictors**

| Predictors | Unstandardised coefficient (B) | Standard Error | Standardised coefficient (β) | 95% CI (lower, upper) |
|---|---|---|---|---|
| SARD (no vs. yes) | -.82 | .33 | -.17* | -1.47, -.17 |
| Other internal chronic disease (no vs. yes) | .12 | .47 | .02 | -.80, 1.04 |

**Note.** n = 221. $R^2$ = .028 (Adjusted $R^2$= .019) and the F for the change in $R^2$ is 3.14 (p=.045). *p<0.05.

## 2. SARD Subgroups

**Model 3: Linear Regression Model predicting the total DASS-21 Score in the Inflammatory Joint group**

| Predictors | Unstandardised coefficient (B) | Standard Error | Standardised coefficient (β) | 95% CI (lower, upper) |
|---|---|---|---|---|
| Difficulties obtaining a rheumatological appointment during the pandemic (no vs. yes) | 12.76 | 4.73 | .37* | 3.18, 22.34 |
| Currently taking anti-rheumatic medication (yes vs. no) | 6.13 | 6.45 | .13 | -6.91, 19.18 |
| Organ involvement (no vs. yes) | 3.78 | 3.22 | .16 | -2.74, 10.31 |
| Active disease within the last 6 months (no vs. yes) | 3.03 | 3.35 | .13 | -3.75, 9.81 |
| Other internal chronic disease (no vs. yes) | 7.49 | 3.57 | .29* | .27, 14.73 |
| Coronavirus Anxiety Score | 1.55 | 1.25 | .17 | -.98, 4.08 |

**Note.** n = 46. $R^2$ = .34 (Adjusted $R^2$= .23) and the F for the change in $R^2$ is 3.18 (p=.013). *p<0.05

**Model 4: Linear Regression Model predicting the total DASS-21 Score in the Systemic Connective Tissue Disease group**

| Predictors | Unstandardised coefficient (B) | Standard Error | Standardised coefficient (β) | 95% CI (lower, upper) |
|---|---|---|---|---|
| Difficulties obtaining a rheumatological appointment during the pandemic (no vs. yes) | 9.68 | 4.42 | .30* | .80, 18.6 |
| Currently taking anti-rheumatic medication (yes vs. no) | 3.52 | 4.74 | .10 | -6.01, 13.04 |
| Organ involvement (no vs. yes) | -.21 | 3.56 | -.01 | -7.44, 7.02 |
| Active disease within the last 6 months (no vs. yes) | 6.78 | 3.59 | .26 | -.43, 14.00 |
| Other internal chronic disease (no vs. yes) | 3.67 | 4.42 | .12 | -5.20, 12.55 |
| Coronavirus Anxiety Score | 1.19 | .87 | .18 | -.555, 2.94 |

**Note.** n = 57. $R^2$ = .22 (Adjusted $R^2$= .12) and the F for the change in $R^2$ is 2.27 (p=.052). *p<0.05

## 3. Covid-19 infection in the past year

**Model 5: Linear Regression Model predicting the total DASS-21 Score with Covid-19 infection in the past year and SARD group membership as predictors**

| Predictors | Unstandardised coefficient (B) | Standard Error | Standardised coefficient (β) | 95% CI (lower, upper) |
|---|---|---|---|---|
| Covid infection in past year | -.70 | 2.76 | -.02 | -6.14, 4.75 |
| SARD (no vs. yes) | -2.76 | 1.72 | -.11 | -6.14, .62 |

**Note.** n = 226. $R^2$ = .01 (Adjusted $R^2$= .003) and the F for the change in $R^2$ is 1.23 (p=.276).

**Model 6: Linear Regression Model predicting the total CAS Score with Covid-19 infection in the past year and SARD group membership as predictors**

| Predictors | Unstandardised coefficient (B) | Standard Error | Standardised coefficient (β) | 95% CI (lower, upper) |
|---|---|---|---|---|
| Covid infection in past year | -.31 | .52 | -.03 | -1.34,.72 |
| SARD (no vs. yes) | -.84 | .33 | -.17* | -1.48,-.20 |

**Note.** n = 225. $R^2$ = .03 (Adjusted $R^2$= .02) and the F for the change in $R^2$ is 3.382 (p=.036).
